# Long-Term Exposure to Concentrated Ambient PM_2.5_ Increases Mouse Blood Pressure through Abnormal Activation of the Sympathetic Nervous System: A Role for Hypothalamic Inflammation

**DOI:** 10.1289/ehp.1307151

**Published:** 2013-11-15

**Authors:** Zhekang Ying, Xiaohua Xu, Yuntao Bai, Jixin Zhong, Minjie Chen, Yijia Liang, Jinzhuo Zhao, Dongyao Liu, Masako Morishita, Qinghua Sun, Catherine Spino, Robert D. Brook, Jack R. Harkema, Sanjay Rajagopalan

**Affiliations:** 1 Davis Heart & Lung Research Institute, The Ohio State University, Columbus, Ohio, USA; 2 Department of Cardiology, East Hospital, Tongji University School of Medicine, Shanghai, PR China; 3 Division of Environmental Health Sciences, Colleges of Medicine and Public Health, The Ohio State University, Columbus, Ohio, USA; 4 The University of Michigan, Ann Arbor, Michigan, USA; 5 Michigan State University, East Lansing, Michigan, USA

## Abstract

Background: Exposure to particulate matter ≤ 2.5 μm in diameter (PM_2.5_) increases blood pressure (BP) in humans and animal models. Abnormal activation of the sympathetic nervous system may have a role in the acute BP response to PM_2.5_ exposure. The mechanisms responsible for sympathetic nervous system activation and its role in chronic sustenance of hypertension in response to PM_2.5_ exposure are currently unknown.

Objectives: We investigated whether central nervous system inflammation may be implicated in chronic PM_2.5_ exposure-induced increases in BP and sympathetic nervous system activation.

Methods: C57BL/6J mice were exposed to concentrated ambient PM_2.5_ (CAPs) for 6 months, and we analyzed BP using radioactive telemetric transmitters. We assessed sympathetic tone by measuring low-frequency BP variability (LF-BPV) and urinary norepinephrine excretion. We also tested the effects of acute pharmacologic inhibitors of the sympathetic nervous system and parasympathetic nervous system.

Results: Long-term CAPs exposure significantly increased basal BP, paralleled by increases in LF-BPV and urinary norepinephrine excretion. The increased basal BP was attenuated by the centrally acting α_2a_ agonist guanfacine, suggesting a role of increased sympathetic tone in CAPs exposure–induced hypertension. The increase in sympathetic tone was accompanied by an inflammatory response in the arcuate nucleus of the hypothalamus, evidenced by increased expression of pro-inflammatory genes and inhibitor kappaB kinase (IKK)/nuclear factor–kappaB (NF-κB) pathway activation.

Conclusion: Long-term CAPs exposure increases BP through sympathetic nervous system activation, which may involve hypothalamic inflammation.

Citation: Ying Z, Xu X, Bai Y, Zhong J, Chen M, Liang Y, Zhao J, Liu D, Morishita M, Sun Q, Spino C, Brook RD, Harkema JR, Rajagopalan S. 2014. Long-term exposure to concentrated ambient PM_2.5_ increases mouse blood pressure through abnormal activation of the sympathetic nervous system: a role for hypothalamic inflammation. Environ Health Perspect 122:79–86; http://dx.doi.org/10.1289/ehp.1307151

## Background

Hypertension is the leading risk factor contributing to global disease burden (Lim et al. 2012). Over the last few decades, a focused scientific effort on modifiable lifestyle risk factors such as sodium intake, diet, obesity, and inactivity has resulted in fundamental insights into how these factors may increase the propensity for hypertension. The link between exposure to pervasive environmental factors such as air pollution and propensity to hypertension has only recently gained attention (Dong et al. 2013).

Controlled studies in humans and animal models have suggested both acute effects and chronic sustained increases in blood pressure (BP) in response to inhaled particulate matter ≤ 2.5 μm in diameter (PM_2.5_) (Brook and Rajagopalan 2009; Brook et al. 2009; Dvonch et al. 2009; Sun et al. 2008; Ying et al. 2009b). We have postulated that the mechanisms for the acute (within hours) BP increase in response to PM_2.5_ exposure likely differ from mechanisms responsible for sustained increases in BP in response to chronic PM_2.5_ exposure (Brook and Rajagopalan 2009; Brook et al. 2010). Imbalanced activation of the autonomic nervous system in particular has been postulated to play a role in the pathogenesis of acute increases in BP in response to PM_2.5_ exposure, primarily on account of the rapidity of the effects and the association with components of heart rate (HR) variability reflective of sympathetic activation (Brook et al. 2009; Cosselman et al. 2012). On the other hand, BP effects in response to chronic PM_2.5_ exposure in controlled-exposure studies in animals has been attributed to a variety of mechanisms, including the production of dysfunctional nitric oxide synthase and the activation of vasoactive mediators (e.g, endothelin-1 and Rho/ROCK), inflammatory cellular infiltration, and vascular remodeling (Brook and Rajagopalan 2009; Kampfrath et al. 2011; Sun et al. 2008; Thomson et al. 2005; Ying et al. 2009b).

In light of accumulating evidence that sympathetic activation may modulate not only acute variations in BP but also could play a dominant role in chronic sustenance of hypertension via such mechanisms as immune activation, renal sodium handling, and vascular remodeling (DiBona 2013), we investigated the importance of sympathetic activation in the genesis of hypertension with chronic concentrated ambient PM_2.5_ (CAPs) exposure.

## Materials and Methods

*Whole-body ambient inhalational CAPs exposures protocol*. The protocol of animal experiments was approved by The Ohio State University Institutional Animal Care and Use Committee, and all the animals were treated humanely and with regard for alleviation of suffering. Animal exposure and the monitoring of exposure atmosphere and ambient aerosol were performed as described previously using a versatile aerosol concentration enrichment system that was modified for long-term exposures (Kampfrath et al. 2011). Briefly, C57BL/6J mice (8-week-old males, *n* = 12/group, 24 in total) were bought from Jackson Laboratory (Bar Harbor, ME, USA) and were housed in standard cages in a mobile trailer with a 12-hr light/12-hr dark cycle, temperatures of 65–75°F, and relative humidity of 40–60%. After 1 week of acclimation, mice were exposed to CAPs or filtered air (FA) in chambers of the Ohio Air Pollution Exposure System for the Interrogation of Systemic Effects at The Ohio State University. All mice, including both FA and CAPs groups, were exposed at exactly the same time. FA-exposed mice received an identical protocol with the addition of a high-efficiency particulate air filter (Pall Life Sciences, East Hills, NY, USA) positioned in the inlet valve to remove CAPs in the filtered air stream, as described previously by Kampfrath et al. (2011). The exposure protocol comprised exposures for 6 hr/day, 5 days/week (no exposure took place during weekends) for a total duration of 6 months from December 2011 to May 2012.

*Sampling and analyses of ambient PM_2.5_ and CAPs in the exposure chamber*. To calculate exposure mass concentrations of ambient PM_2.5_ and CAPs in the exposure chambers, samples were collected weekly on Teflon filters [Teflo, 37-mm, 2-μm pore (Pall Life Sciences, Ann Arbor, MI, USA)] and weighed before and after sampling in a temperature- and humidity-controlled weighing room using a Mettler Toledo no. 11106057 microbalance (Mettler Toledo, Columbus, OH, USA). Ambient PM_2.5_ and CAPs samples collected on Teflon filters were wetted with ethanol and extracted in 1% nitric acid solution. The extraction solution was sonicated for 48 hr in an ultrasonic bath and then allowed to passively acid digest for a minimum of 2 weeks. We then analyzed sample extracts for a suite of trace elements using inductively coupled plasma-mass spectrometry (ICP-MS) (ELEMENT2; Thermo Finnigan, San Jose, CA, USA) (Morishita et al. 2004).

**Figure 1:**
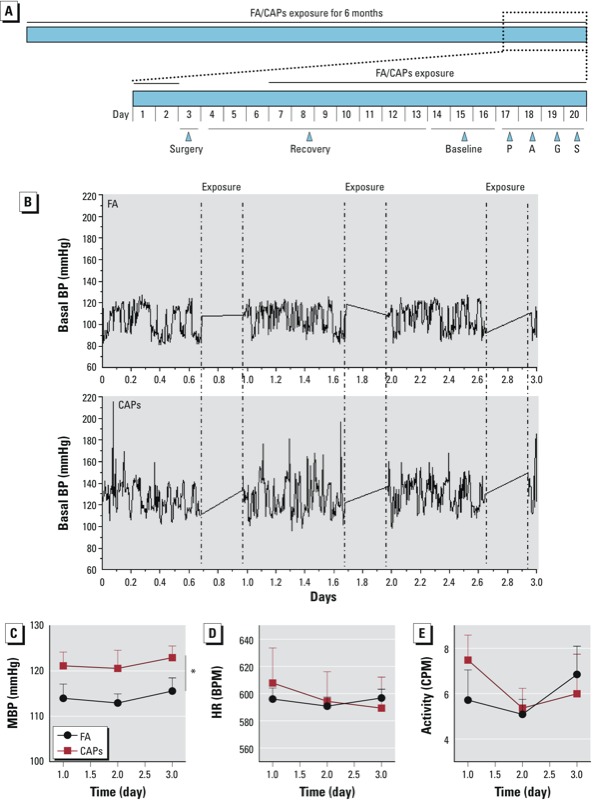
CAPs exposure increases basal BP in C57BL/6J mice. (*A*) Diagram of the experimental time scheme. Abbreviations: A, atropine; G, guanfacine; P, propranolol; S, air-jet stress. After exposure to FA or CAPs for 6 months, mice (*n* = 6/group) were implanted with DSI radiotelemetry transmitters, and the basal BP was analyzed after recovery from surgery. (*B*) Representative recordings: the basal BP was recorded after daily FA or CAPs exposure for approximately 18 hr/day for 3 continuous days. Quantization of 16-hr MAP (1700–0900 hours) (*C*), HR (*D*), and mouse activity (*E*). *p < 0.05, compared with FA using two-way ANOVA.

*BP, HR, and locomotor activity measurement*. FA- and CAPs-exposed C57BL/6J mice (*n* = 6/group) were implanted with DSI radiotelemetric transmitters (model TA11PA-C10; Data Sciences International, St. Paul, MN, USA) as described previously by Tallam et al. (2005). To prevent the confounding effects of surgery, we allowed the mice to recover for 10 days after surgery (with no exposure to CAPs in the first 4 days). By 10 days after surgery, the mice had regained their circadian BP and HR and the surgery- and anesthesia-dependent initial changes in BP and HR had been replaced with stable values (Figure 1B). We then began monitoring mean arterial pressure (MAP) and HR as outlined in Figure 1A. Basal MAP, HR, and locomotor activity were recorded for 3 days (18 hr/day) Figure 1C–E). The typical MAP, HR, and locomotor activity data in a 16-hour period (1700–0900 hours) were analyzed and are presented here.

To evaluate autonomic control of BP, the following drugs were applied as described previously by Gross et al. (2005): the muscarinic blocker atropine (2 mg/kg), β-adrenergic receptor blocker propranolol (4 mg/kg), and α_2_-adrenergic agonist guanfacine (1 mg/kg). To prevent potential interference by the preceding drug administration, mice were allowed to rest for at least 22 hr before their next drug treatment. All drugs were administered intraperitoneally between 1600 and 1800 hours. Continuous beat-by-beat values of BP were recorded for 30 min before and 2 hr after administration of drugs. As described previously by Gross et al. (2005), the values from the 45th to the 60th minute after drug injection were used to characterize the responses to the drugs and to avoid measuring stress-induced BP and HR changes.

To determine whether chronic CAPs exposure alters the MAP and HR response to an acute pressor stress, we utilized a previously described model of acute stress that combines restraint with pulsatile, unavoidable bursts of air to the forehead for 1 min (do Carmo et al. 2011). Briefly, all mice were acclimatized to the restrainer tube for 1 hr before actual experimentation to minimize trauma associated with entry into the restrainer tubes. BP was monitored by telemetry for 15 min. Air-jet stress consisted of pulses (2 sec in duration delivered every 10 sec for 1 min) of compressed air (15 lb/​in^2^) aimed at the forehead from an opening at the front of the tube. After the 1-min air-jet stress, MAP was measured for an additional 30-min recovery period. BP response during the air-jet stress and recovery period after the air-jet stress were calculated as the changes compared with baseline period measurements. We also calculated the areas under the MAP curve during the air-jet stress and recovery periods.

*Spectral analysis*. We calculated the power spectra of systolic BP, pulse interval time series, and the cross spectra using fast Fourier transform (FFT). Beat-to-beat values of detected R-R intervals and BP values were interpolated, low-pass filtered (cutoff 6 Hz), and resampled at 12 Hz. Data segments of 43 sec were used for spectral analysis. Linear trends were removed, and we estimated power spectral density with the FFT-based Welch algorithm using segments of 512 data points with 50% overlapping and Hanning window. We calculated the power in the low frequency range (LF, 0.25–0.6 Hz). Five representative intervals were chosen for spectral analysis by a researcher who did not know the treatment of the animals, and the intervals were averaged according to the following criteria: *a*) steady-state conditions, *b*) no large sudden BP changes, and *c*) no artifacts.

*Myograph*. The mesenteric bed was removed from mice exposed to FA or CAPs, as described above [but not subjected to telemetric transmitter implantation surgery or subsequent analysis (6/group)] and euthanized 1 week later than other mice. Mesenteric arteries (2-mm segments of the second-order branch of the superior mesenteric artery) were dissected free of fat and connective tissue and mounted in wire myograph chambers (DMT 620M; Aarhus N, Denmark). The arterial vessel segments were maintained at 37°C in physiological Krebs’ buffer that was bubbled with 95% oxygen/5% carbon dioxide to maintain the buffer at pH 7.4. After a 30-min equilibration period, vessel tension was increased to 1 mN. A further 30 min after resting tension was established, mesenteric arteries were maximally contracted with a potassium ion-depolarizing solution [KPSS (123 mmol/L postassium chloride, 1.17 mmol/L mg sulfate, 2.37 mmol/L potassium dihydrogen phosphate, 2.5 mmol/L calcium chloride, 11.1 mmol/L d-glucose, and 0.026 mmol/L EDTA)]. The vessels were subjected to graded doses of the vasoconstrictors phenylephrine (10^−9^ to 10^−5^ mol/L) and U46619 (10^−8^ to 10^−6.5^ mol/L). The results were expressed as a percentage of contraction by 120 mm. To test the acetylcholine-induced vasodilatation, mesenteric arterial rings were precontracted by phenylephrine (1 μm). After a stable contraction plateau was reached with phenylephrine, the vessels were exposed to the endothelium-dependent vasodilator actylcholine (10^−8^ to 10^−5^ mol/L). Results were expressed as a percentage of precontraction by phenylephrine.

*Urine collection and noradrenaline analysis*. We collected urine during the week before implantation of telemetry transmitters. After the daily exposure, mice (6/group) were placed into urine collection cages. The urine was then collected the next morning. Every mouse was subject to two separate collections, and the urine from the same animal was pooled and stored at –80^o^C until analysis. The concentration of noradrenaline in urine was analyzed with the norepinephrine ELISA kit (Abnova, Taipei City, Taiwan) according to the manufacturer's instructions.

*Immunofluorescence analysis*. A subgroup of the FA- and CAPs-exposed mice that were used in the myograph experiments above and used for immunofluorescence analysis were also anesthetized with isoflurane and blood was collected through the large retroorbital plexus before the mice were euthanized in the week after the completion of BP analysis. After ligating and separating the thoracic aorta with a hemostat, perfusion was performed with 30 mL phosphate buffered saline (PBS) via intracardiac injection. The vasculature was then pre-fixed through perfusion of 15 mL paraformaldehyde (4%). Brains were removed rapidly and fixed at 4°C for 1 day in paraformaldehyde (4%) then cryoprotected in 30% sucrose-PBS for 3 days, frozen, and sectioned in 6-μm-thick coronal slices. Inhibitor kappaB kinase (IKK) phosphorylation and c-fos expression were performed according to the protocols described previously by Purkayastha et al. (2011a). Both rabbit phospho-IKK (Ser176/180) and c-fos antibodies were purchased from Cell Signaling (EMD Millipore, Billerica, MA, USA).

*Quantitative real-time reverse transcription polymerase chain reaction (RT-PCR)*. We isolated total RNA of hypothalamus (from the same animals as described above in “BP, HR, and locomotor activity measurement”) with TRIzol reagent (Invitrogen, Carlsbad, CA, USA). We reverse transcribed 4 μg total RNA by random hexamers and the ThermoScript RT-PCR System (Invitrogen). Quantitative RT-PCR was performed with the Stratagene Mx3005 using SYBER Green PCR Master Mix (Applied Biosystems, Carlsbad, CA, USA). The sequences of primers used were as follows: glyceraldehyde-3-phosphate dehydrogenase (GAPDH): sense, 5´-TGA ACG GGA AGC TCA CTG G-3´, antisense, 5´-TCC ACC ACC CTG TTG CTG TA-3´; tumor necrosis factor-α (TNFα): sense, 5´-GGC ACC ACT AGT TGG TTG TCT TTG-3´, antisense, 5´-AGA AAT GCA GTC AGC ACC ATC AAG-3´; interleukin-6 (IL-6): sense, 5´-TGA TGC TGG TGA CAA CCA CGG C-3´, antisense, 5´-TAA GCC TCC GAC TTG TGA AGT GGT A-3´); E-selectin: sense, 5´-GGC CAG CGC AGG TTG AAT GC-3´, antisense, 5´-ATG TTG CCC TGC TGT GGC GC-3´; intercellular adhesion molecule 1 (ICAM-1): sense, 5´-CCG GTC CTG ACC CTG AGC CA-3´, antisense, 5´-ATT GGA CCT GCG GGG TGG GT-3´; IL-10: sense, 5´-CAC AAA GCA GCC TTG CAG AA-3´, antisense, 5´-CTG GCC CCT GCT GAT CCT-3´; and vascular cell adhesion molecule 1 (VCAM-1): sense, 5´-GGA GAC CTG TCA CTG TCA ACT G-3´, antisense, 5´-TCC ATT TCA CCA CTG TGT AAC C-3´. The relative expression level was obtained as described previously by Ying et al. (2009a).

*Statistical analysis*. All data are expressed as means ± SDs unless noted otherwise. Statistical tests were performed using one-way or two-way analysis of variance (ANOVA) followed by Bonferroni correction or unpaired *t*-test using GraphPad Prism (version 4.1.2; GraphPad Software, La Jolla, CA, USA). The significance level was set at *p* < 0.05.

## Results

*CAPs exposure data*. The average ambient daily PM_2.5_ concentration during the period was 9 ± 1 μg/m^3^. The average CAPs concentration in CAPs chambers was 107 ± 6 μg/m^3^ versus 3 ± 1 μg/m^3^ in FA chambers. The exposures were performed for 6 hr/day, 5 days/week, and the normalized daily CAPs concentration was 26.5 μg/m^3^, which was significantly higher than the annual national ambient air quality standard of 12 μg/m^3^ set by the U.S. Environmental Protection Agency (U.S. EPA 2012). Table 1 shows the elemental composition of the ambient PM_2.5_ and CAPs during the exposure period. Black carbon in ambient PM_2.5_ and CAPs during the exposure period was 492.5 ± 283.4 and 5,269.5 ± 2,616.2 ng/m^3^, respectively. Among all chemical components in Table 1, sulfur and selenium had the two highest correlations with PM_2.5_ mass (0.94 and 0.84, respectively). Although identifying PM_2.5_ emission sources is beyond the scope of this paper, the chemical characterization data showed that the study site was most strongly affected by secondary aerosols, which are likely to include emissions from coal-fired utility boilers located regionally (Reff et al. 2009).

**Table 1:** Elemental composition of ambient PM_2.5_, FA, and CAPs [mean ± SD (ng/m^3^)].

Element	Ambient PM_2.5_	FA	CAPs
Rubidium	0.07 ± 0.02	0.03 ± 0.02	0.66 ± 0.23
Strontium	0.37 ± 0.09	0.12 ± 0.04	3.81 ± 1.28
Molybdenum	0.33 ± 0.12	0.13 ± 0.11	2.93 ± 1.35
Cadmium	0.24 ± 0.09	0.5 ± 0.37	1.8 ± 1.02
Antimony	0.68 ± 0.25	0.06 ± 0.04	6.39 ± 2.84
Barium	2.41 ± 0.59	0.65 ± 0.21	25.92 ± 9.24
Lanthanum	0.03 ± 0.01	0	0.33 ± 0.18
Cerium	0.04 ± 0.01	0.02 ± 0.01	0.44 ± 0.21
Lead	2.8 ± 0.62	2.06 ± 0.78	23.3 ± 6.14
Sodium	54.37 ± 25.62	73.46 ± 25.17	453.74 ± 303.19
Magnesium	18.72 ± 4.69	7.76 ± 2.85	202.36 ± 61.84
Aluminum	22.89 ± 8.09	19.77 ± 13.38	201.34 ± 68.75
Phosphorus	10.2 ± 2.15	17.55 ± 3.1	64.72 ± 10.42
Sulfur	790.17 ± 455.19	33.69 ± 16.58	7556.13 ± 4759.81
Calcium	70.95 ± 19.94	74 ± 39.23	698.49 ± 220.44
Titanium	0.82 ± 0.17	0.1 ± 0.04	9 ± 2.33
Vanadium	0.24 ± 0.15	0.02 ± 0.01	2.25 ± 1.47
Chromium	3.22 ± 0.48	6.86 ± 0.99	13.59 ± 1.54
Manganese	1.92 ± 0.82	0.18 ± 0.15	19.11 ± 9.57
Iron	43.26 ± 12.69	11.59 ± 10.21	462.3 ± 145.59
Cobalt	0.09 ± 0.1	0.11 ± 0.1	0.81 ± 1.02
Nitrogen	0.38 ± 0.43	0.42 ± 0.44	2.61 ± 1.43
Copper	2.33 ± 0.62	1.58 ± 1.42	23.98 ± 7.57
Zinc	10.3 ± 3.17	6.81 ± 5.66	90.07 ± 29.94
Potassium	34.96 ± 11.13	22.49 ± 8.11	313.02 ± 103.66
Arsenic	0.56 ± 0.19	0.03 ± 0.01	5.26 ± 2.31
Selenium	0.74 ± 0.28	0.05 ± 0.02	7.27 ± 3.59
PM_2.5_ was collected weekly, and the average during the 6 months of exposure is presented.			

*CAPs exposure increases mouse BP*. All animals were weighed before sacrifice. We found no significant difference in body weight between FA- and CAPs-exposed groups postexposure (34.7 ± 2.3 and 32.5 ± 2.9 g, FA and CAPs, respectively). As shown in Figure 1B and 1C, CAPs exposure significantly increased basal MAP. In contrast, exposure did not significantly induce changes in HR (Figure 1D) or locomotor activity (Figure 1E).

**Figure 2:**
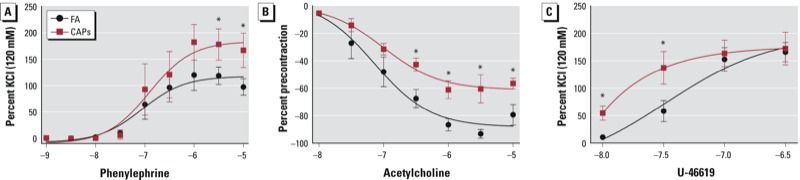
CAPs exposure induces resistance arterial dysfunction. After sacrifice, mouse mesenteric arteries were isolated and mounted onto a wire myograph, and responses to phenylephrine (*A*), acetylcholine (*B*), and U-46619 (*C*) were analyzed. *p < 0.05, compared with FA using two-way ANOVA.

*CAPs exposure induces vascular dysfunction*. Because resistance, not conduit, arteries primarily determine total peripheral resistance and BP, we isolated mesenteric arteries and assessed their functional response in mice that had undergone the same exposure but were not subject to intra-arterial catheter placement. Figure 2 reveals that CAPs exposure significantly increased the contractile response of mesenteric arteries to phenylephrine (a selective α_1_-adrenergic receptor agonist) and U-46619 (a thromboxane A2 receptor agonist), and reduced relaxation responses to acetylcholine (an endothelium-dependent vasodilator). LogEC_50_ (concentration of a drug at which half-maximal response is observed) and peak responses are enumerated in Table 2. However, the aortic mRNA expression of α_1_ adrenergic receptors was not changed by CAPs exposure (see Supplemental Material, Figure S1).

**Table 2:** The log_EC50_s and peak responses (means ± SDs) of mesenteric arteries from FA- and CAPs-exposed mice.

	logEC_50_		Peak response	
	FA	CAPs	FA	CAPs
Phenylephrine	–7.1 ± 0.2	–6.9 ± 0.2	117.3 ± 11.3	183.8 ± 18.7*
Acetylcholine	–7.1 ± 0.2	–7 ± 0.2	–88.3 ± 4.6	–61.1 ± 3.7*
U-46619	–7.5 ± 0.3	–9.4 ± 0.9*	197.3 ± 27	181.1 ± 24
*p < 0.05 compared with FA, using Student’s *t*-test.				

**Figure 3:**
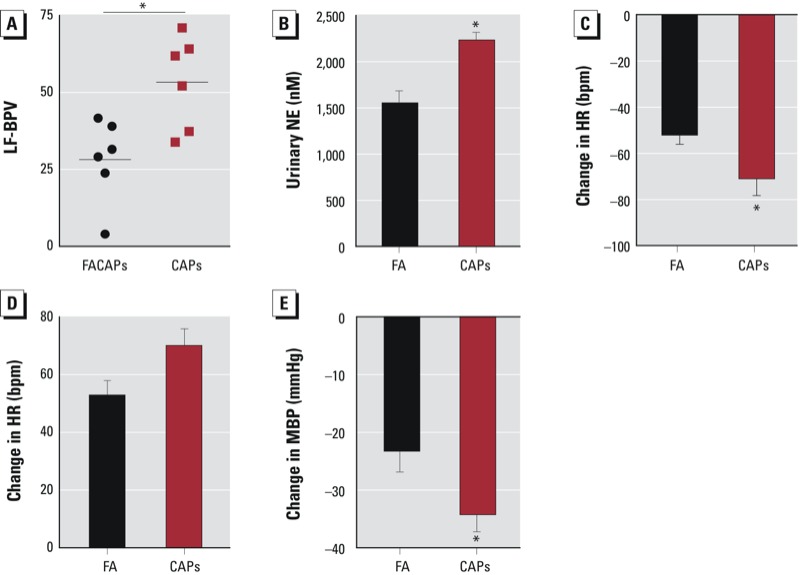
CAPs exposure increases sympathetic tone. (*A*) The LF-BPV was calculated from the 3 days of basal BP recording. (*B*) Urine of FA- and CAPs-exposed mice was collected and norepinephrine excretion was assessed. After analysis of the basal BP in FA- and CAPs-exposed mice with radiotelemetry transmitter, mice were treated with propranolol (*C*), atropine (*D*), and guanfacine (*E*), and changes in either HR (propranolol and atropine) or BP (guanfacine) is presented. NE, norepinephrine. *p < 0.05, compared with FA using Student’s *t*-test.

*CAPs exposure activates the sympathetic nervous system*. Abnormal activation of the sympathetic nervous system has been implicated in the pathogenesis of human hypertension (Paton et al. 2013). We observed increased BP variability in CAPs-exposed animals (lower panel of Figure 1B). Because low-frequency variation of BP (LF-BPV) is an index of sympathetic tone, we assessed these changes in response to exposure. Figure 3A reveals that CAPs exposure significantly increased LF-BPV. We also analyzed catecholamine production by measuring excretion of the sympathetic neurotransmitter norepinephrine. As shown in Figure 3B, CAPs exposure significantly increased urine excretion of norepinephrine. In contrast, we found no significant difference in collected urine volume (FA vs. CAPs: 0.5 ± 0.2 vs. 0.4 ± 0.2 mL, *n* = 6, *p* = 0.6, Student’s *t*-test)

Consistent with the increased sympathetic tone in response to long-term CAPs exposure, a nonselective beta-blocker, propranolol, significantly decreased HR in CAPs-exposed but not in FA-exposed animals (Figure 3C; see also Supplemental Material, Table S1). In contrast, atropine did not induce significant HR changes in either FA-exposed or CAPs-exposed animals (Figure 3D; see also Supplemental Material, Table S1). We observed no significant effects on BP as a result of either propranolol or atropine exposure. This is consistent with previous reports by Rodrigues et al. (2011) and Fazan et al. (2005) that neither propranolol nor atropine induces significant BP responses in various situations. To assess whether the increased sympathetic tone in CAPs-exposed animals contributes to hypertension, we acutely inhibited the sympathetic nervous system with a selective α_2a_ receptor agonist, guanfacine. Figure 3E illustrates that guanfacine administration resulted in a significantly larger drop in BP in CAPs-exposed animals.

**Figure 4:**
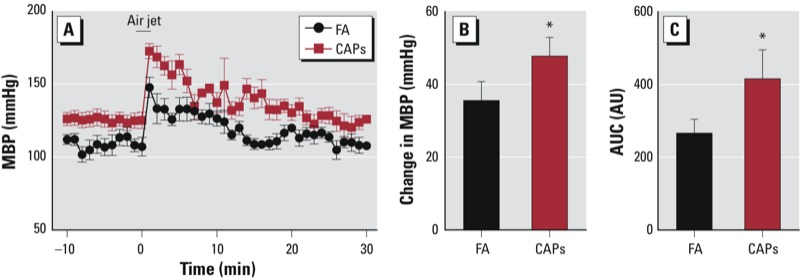
CAPs exposure increases stress-induced hypertension. FA- and CAPs-exposed mice were stimulated with air jet, and BP response was analyzed with radiotelemetry transmitter. (*A*) The BP response curve. (*B*) The peak increase in BP. (*C*) The area under the BP response curve (AUC). AU, arbitrary units. *p < 0.05, compared with FA using Student’s *t*-test.

*CAPs exposure increases the hypertensive response to air-jet stress*. Air-jet stress typifies psychoemotional stress and invokes a characteristic “defense reaction” that is typified by an autonomic nervous system–mediated increase in MAP. Figure 4 shows that, compared with control mice, CAPs-exposed mice had a significantly increased peak BP response to air-jet stress, and the area under the MAP curve was also significantly increased.

**Figure 5:**
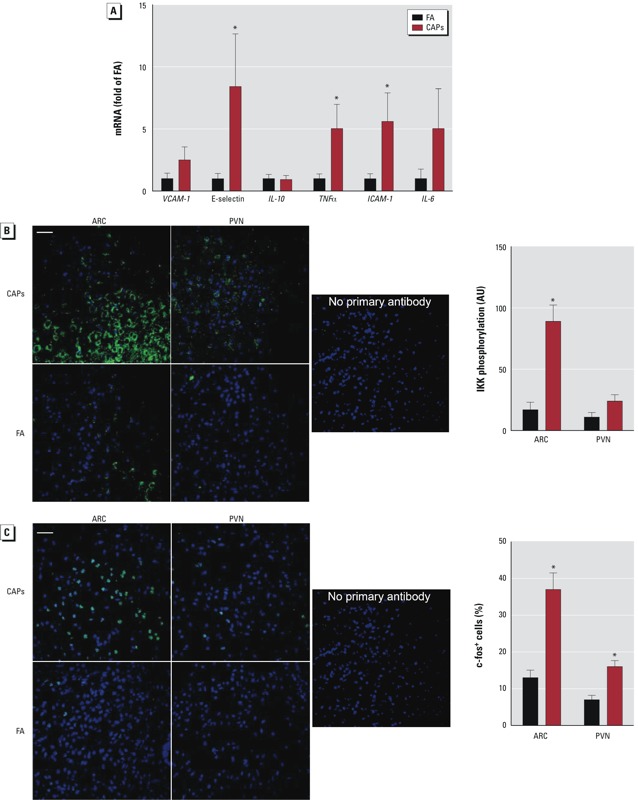
CAPs exposure induces hypothalamic inflammation. (*A*) FA- and CAPs-exposed mice were sacrificed, and hypothalamic expression of pro-inflammatory genes was analyzed by RT-PCR. Immunostaining of mouse hypothalamus with anti-phospho-IKK2 (*B*) and anti-c-fos (*C*). Abbreviations: ARC, arcuate nucleus; AU, arbitrary units; PVN, paraventricular nucleus. In *B* and *C*, bar = 25 μm. *p < 0.05, compared with FA using Student’s *t*-test.

*CAPs exposure induces hypothalamic inflammation*. An inflammatory response in the hypothalamus has been shown to play a critical role in the activation of the sympathetic nervous system in response to diverse pathological stimuli and to mediate the obesity-induced increase in BP (Kang et al. 2009; Purkayastha et al. 2011a; Yu et al. 2012). We therefore isolated mouse hypothalamus and assessed the expression of pro-inflammatory genes by RT-PCR. CAPs exposure significantly increased hypothalamic mRNA expression of endothelial-leukocyte adhesion molecule 1 (E-selectin), TNFα, and ICAM-1 (Figure 5A). The IKK/NF-kappaB (NF-κB) signaling pathway is central in inflammatory responses (Gamble et al. 2012). Figure 5B reveals that similar to the response to a high-fat diet (Purkayastha et al. 2011a), the phosphorylation of IKK in mouse hypothalamic arcuate nucleus was markedly increased in response to chronic CAPs exposure. In contrast, long-term CAPs exposure induced only a nonsignificant trend toward an increase in IKK phosphorylation in the hypothalamic paraventricular nucleus. Dragunow and Faull (1989) previously demonstrated that the expression of c-fos, an indirect marker of neuronal activity, is markedly increased in response to CAPs exposure in the hypothalamic arcuate nucleus. Figure 5C shows that consistent with the level of IKK phosphorylation, long-term CAPs exposure also significantly increased the number of c-fos–positive cells in the mouse hypothalamic paraventricular nucleus.

## Discussion

Our findings demonstrate that *a*) long-term CAPs exposure significantly increased BP in C57BL/6J mice, paralleled by marked alterations in resistance vessel tonal responses to agonist stimulation; *b*) long-term CAPs exposure induced hypertension and abnormal activation of the sympathetic nervous system, as evidenced by increased LF-BPV, increased urinary norepinephrine excretion, and attenuation of BP with guanfacine; and *c*) sympathetic nervous system activation is paralleled by an inflammation in the hypothalamus, suggesting that the latter may play a permissive role in chronic CAPs exposure–induced hypertension.

Epidemiologic and empirical evidence strongly support the hypothesis of short-term effects of air pollution on the cardiovascular system, with a magnification of these effects with chronic exposure (Brook et al. 2010; Miller et al. 2007). This is consistent with the concept that chronic and cumulative exposure to air pollution occurring over an individual's lifetime should result in more pronounced effects, perhaps as a result of sustained effects due to particle exposure (Miller et al. 2007). From a BP perspective, models that provide a unified understanding of short-term effects, that also help reconcile chronic effects of PM_2.5_ exposure on hypertension and their mechanisms, are needed. Acute exposure to both PM_2.5_ and diesel exhaust particles triggers rapid changes in BP associated with abnormalities in HR variability indices traditionally associated with sympathetic activation (Brook et al. 2009; Cosselman et al. 2012). BP effects in response to chronic CAPs exposure in animals, on the other hand, have been attributed to a variety of mechanisms, including production of dysfunctional nitric oxide synthase and vasoactive mediators such as endothelin-1 as well as Rho/ROCK activation, inflammatory cellular infiltration in the perivascular adipose tissue, and vascular remodeling (Brook and Rajagopalan 2009). The notion that sympathetic nervous system may modulate chronic hypertension through inflammation in key regulatory areas in the central nervous system represents a paradigm shift in our understanding of hypertension (Andersson and Tracey 2012; Elenkov et al. 2000; Harrison et al. 2011; Leibowitz and Schiffrin 2011; Olofsson et al. 2012). This has changed our view of the autonomic nervous system as an arbitrator of acute changes in HR and BP to a facilitator of chronic functional and structural changes in hypertension (Abboud et al. 2012; Harrison et al. 2011). Studies from several research groups suggest that diverse stimuli, including stress, may lead to modest elevations in BP (to a pre-hypertensive state) via sympathetic nervous system mechanisms (e.g., Marvar et al. 2011). This early phase may lead to a more protracted chronic phase driven by inflammatory mechanisms, further leading to a perpetuation of hypertension. Thus the sympathetic nervous system appears to mediate both short-term changes in BP and also pathways that may lead to perpetuation of hypertension (Abboud et al. 2012). Our results suggest that chronic exposure to PM_2.5_ may represent yet another factor leading to chronic sympathetic activation and hypertension. The increase in sympathetic tone in response to chronic PM_2.5_ exposure is supported by findings of increased LF-BPV, heightened urinary norepinephrine excretion, and abnormal hypertensive responses to air-jet stress (a model of hypertension associated with sympathetic activation). Furthermore, the central sympathetic inhibitor guanfacine ablated the BP response to chronic CAPs exposure. Sympathetic nervous system activation in the central nervous system is accomplished via alteration of presynaptic α_2_-adrenergic receptors, which are critical in determining central catecholamine levels and activation. The adrenergic α_2a_ receptors are almost exclusively expressed in the central nervous system and have sympathoinhibitory effects, whereas α_2b_ has a sympathoexcitatory function (MacMillan et al. 1996; Makaritsis et al. 1999, 2000). The effects of guanfacine, but not propranol or atropine, in lowering BP suggest that central sympathetic nervous system activation likely plays an important role in the effects of PM_2.5_ on BP.

There are emerging data that hypothalamic inflammation plays an important role in regulation of sympathetic tone in response to diverse pathophysiological stimuli such as a high-fat diet, angiotensin II, and heart failure (Kang et al. 2009; Purkayastha et al. 2011b; Yu et al. 2012). Interestingly, MohanKumar et al. (2008) showed that chronic CAPs exposure increased NF-κB activation and pro-inflammatory gene expression paralleled by an increase of neurotransmitter norepinephrine in the hypothalamic paraventricular nucleus. Our data, although somewhat consistent with those findings, also indicate increased expression of pro-inflammatory genes and IKK activation in the arcuate nucleus with less activation in the paraventricular nucleus. Importantly, the local increase in IKK activation was correlated to neural activity in these locations, as evidenced by the increased number of c-fos–positive cells. Although these are suggestive of a role for inflammation in increased sympathetic tone and consequent hypertension in response to chronic PM_2.5_ exposure, definitive proof may require additional experiments such as disruption of the IKK/NF-κB signaling pathway in the hypothalamus and assessment of how this affects the chronic PM_2.5_ exposure–induced increase in BP. An additional interpretative difficulty is the relative importance of specific circumventricular organs that may differentially contribute to hypertension. For instance, POMC (proopiomelanocortin) neurons have been shown to be involved with high-fat diet–mediated BP effects, whereas AgRP (agouti-related peptide) neurons have not (Purkayastha et al. 2011b). Further, inflammation in sites such as the nucleus tractus solitarius and other vagal centers (the dorsal ventral lateral medulla and the nucleus ambiguus) may also play a role (Teslovich et al. 2010; Waki et al. 2011). In several models of hypertension, enhanced activation of circuits involving γ-aminobutyric acid and increased glutamate receptor activation in the paraventricular nucleus and rostral ventrolateral medulla, contribute to the elevated sympathetic nervous system activity and BP. In this regard, it is interesting to postulate that air pollution may activate both rapid and slow central nervous system pathways regulating sympathoexcitation.

Although our results provide evidence that chronic CAPs exposure increases sympathetic tone and BP, CAPs exposure did not increase HR. This is consistent with previous studies showing that acute CAPs exposure, while elevating BP, did not affect cardiac output in either humans (Brook et al. 2009) or dogs (Bartoli et al. 2009). Although the reasons for the selective modulation of presser responses without alterations in HR are unclear at this time, one potential explanation may relate to the fact that sympathetic control may be differentially modulated in various organs in response to diverse stimuli (do Carmo et al. 2011; Esler et al. 2006). Thus, the effects of CAPs exposure on sympathetic tone may also be organ specific. Therefore, it is important in the future to assess the sympathetic tone of different organs, in particular those central in BP regulation, such as the kidney.

After normalization, CAPs exposure level was still higher than the annual national ambient air quality standard set by the U.S. EPA (2012). Although these mean exposure levels are currently unusual in North America, they are not rare in cities with heavy air pollution. For instance in 2005, 89% of the world’s population lived in areas where the World Health Organization (WHO) Air Quality Guideline of 10 μg/m^3^ PM_2.5_ (annual average) was exceeded (Brauer et al. 2012). Globally, 32% of the population lived in areas exceeding the WHO Level 1 Interim Target of 35 μg/m^3^, driven by high proportions in East and South Asia (76% and 26%, respectively) (Brauer et al. 2012).

Our study has a number of important limitations. These include the fact that we have not provided a cause-and-effect relationship between inflammation in the hypothalamus, sympathetic nervous system activation, and BP responses. This will require additional experiments targeting inflammation in the hypothalamus and concomitantly measuring sympathetic nervous system activation and BP. We have not provided any data on the time course of sympathetic nervous system activation, inflammation, and BP response. In addition, our interventional experiments with sympathetic and parasympathetic blockade were performed acutely, and it remains to be seen if chronic blockade with guanfacine attenuates the development of BP in response to CAPs. Another limitation is the fact that we have not done comparative assessment of other areas of the brain such as the nucleus tractus solitarius and centers such as the dorsal ventral lateral medulla and the nucleus ambiguous, which may also play a role in BP responses to CAPs exposure (Teslovich et al. 2010; Waki et al. 2011).

## Conclusion

Our data provide evidence that increased sympathetic tone mediates the hypertensive action of chronic CAPs exposure, which may involve an inflammatory response in the hypothalamus.

## Supplemental Material

(258 KB) PDF
