# Supplementary material for: Long-Term Exposure to Concentrated Ambient PM2.5 Increases Mouse Blood Pressure through Abnormal Activation of the Sympathetic Nervous System: A Role for Hypothalamic Inflammation
Source: Environ Health Perspect. 2013 Nov 15;122(1):79–86. doi: 10.1289/ehp.1307151 (PMC3888575; doi:10.1289/ehp.1307151)
Supplement: (258 KB) PDF [file ehp.1307151.s001.508.pdf]

Supplemental Material

Long-Term Exposure to Concentrated Ambient $PM_{2.5}$ Increases Mouse Blood Pressure through Abnormal Activation of the Sympathetic Nervous System: A Role for Hypothalamic Inflammation

Zhekang Ying, Xiaohua Xu, Yuntao Bai, Jixin Zhong, Minjie Chen, Yijia Liang, Jinzhuo Zhao, Dongyao Liu, Masako Morishita, Qinghua Sun, Catherine Spino, Robert D. Brook, Jack R. Harkema, Sanjay Rajagopalan

Table of Contents

Supplemental Material, Table S1---2

Supplemental Material, Figure S1--3

Reference---4

Supplemental Material, Table S1. HR and BP responses to ANS inhibitors (Mean ± SD).

		Propranolol		Atropine		Guanfacine	
Parameter	Exposure	Pre	Post	Pre	Post	Pre	Post
HR (BPM)							
	FA	575 ± 66	503 ± 59	571 ± 88	624 ± 79	589 ± 87	497 ± 49#
	CAPs	593 ± 81	489 ± 47*	603 ± 86	673 ± 97	576 ± 79	483 ± 78#
BP (mmHg)							
	FA	113 ± 8	107 ± 6	116 ± 7	117 ± 6	116 ± 10	93 ± 6#
	CAPs	124 ± 6*	123 ± 12*	125 ± 11	133 ± 14*	113 ± 9*	97 ± 11#

FA- and CAPs-exposed mice were treated with ANS inhibitors. The mean blood pressure (MBP) and heart rate (HR) responses to drug administration were analyzed by telemetry as described in Methods. N=6. *p<0.05 vs FA, #p<0.05 vs Pre, two way ANOVA.

Supplemental Material, Figure S1.

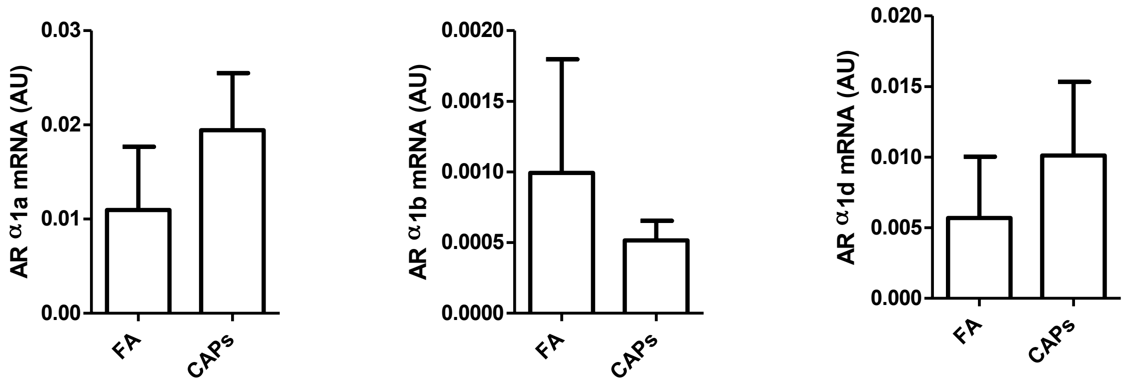

The total RNA was isolated from aorta (from the same animals used for blood pressure analysis), and the mRNA expression level of adrenergic receptors (AR) was analyzed by real-time RT-PCR as described previously (Cikos et al 2007).

Reference

Cikos S, Rehák P, Czikková S, Veselá J, Koppel J. 2007. Expression of adrenergic receptors in mouse preimplantation embryos and ovulated oocytes. Reproduction. 133(6):1139-1147.
